# Endogenous Retrovirus ev21 Dose Not Recombine with ALV-J and Induces the Expression of ISGs in the Host

**DOI:** 10.3389/fcimb.2016.00140

**Published:** 2016-10-25

**Authors:** Min Feng, Yan Tan, Manman Dai, Yuanfang Li, Tingting Xie, Hongmei Li, Meiqing Shi, Xiquan Zhang

**Affiliations:** ^1^Department of Animal Genetics, Breeding and Reproduction, College of Animal Science, South China Agricultural UniversityGuangzhou, China; ^2^Guangdong Provincial Key Lab of Agro-Animal Genomics and Molecular Breeding and Key Lab of Chicken Genetics, Breeding and Reproduction, Ministry of AgricultureGuangzhou, China; ^3^Department of Preventive Veterinary, College of Veterinary Medicine, South China Agricultural UniversityGuangzhou, China; ^4^Division of Immunology, Virginia-Maryland Regional College of Veterinary Medicine, University of MarylandCollege Park, MD, USA

**Keywords:** *ev21*, late feathering chicken, ALV-J, ISGs, envelope protein

## Abstract

Avian leukosis virus subgroup J (ALV-J) infection can cause tumors and immunosuppression. Endogenous viruses integrate into host genomes and can recombine with exogenous avian leukosis virus (ALV). In this study, we analyzed the interaction of endogenous retrovirus 21 (*ev21*) with the ALV-J in late-feathering Chinese yellow chicken. Two ALV-J strains M180 and K243 were isolated from late-feathering and fast-feathering Chinese yellow chicken flocks, respectively. The *env* gene of the two strains showed 94.2–94.8% nucleotide identity with reference ALV-J strains. Compared with the *env* gene and the LTR of *ev21* and M180, the nucleotide identity of LTR was 69.7% and *env* gene was 58.4%, respectively, especially the amino acid identity of *env* gene as low as 14.2%. Phylogenetic analysis of the nucleotide sequence of the env gene and the 3′LTR showed that M180 was closely related to ALV-J, and was located in a distinct group with *ev21* in the phylogenetic tree. Using co-immunoprecipitation (co-IP), we next demonstrate that the envelope protein of *ev21* does not interact with the M180 envelope protein. We further show that the envelope protein of *ev21* cannot activate ALV-J LTR promoter activity using luciferase-reporter assays. qPCR and western blot analysis revealed that envelope protein of endogenous *ev21* can facilitate the expression of *PKR* at 6h post ALV-J infection (hpi) and facilitate the expression of *ISG12* and *CH25H* at 24 hpi. However, the expression of the *env* gene of M180 strain was not significantly at 6 and 24 hpi. We conclude that there is no evidence of recombination between endogenous retrovirus ev21 and ALV-J strain M180 in late-feathering Chinese yellow chicken, and envelope protein of ev21 can affect the expression of host ISGs, but appears not to influence the replication of ALV-J strain M180. This is the first report of interaction among the endogenous retrovirus ev21, ALV-J and the late-feathering chicken.

## Introduction

The late- and fast-feathering phenotypes of chicken are widely utilized in poultry production to identify the sex of chicks at hatching. This phenotype is determined by K loci located on the Z chromosome (Elferink et al., 2008). It has been found that the avian endogenous retrovirus gene 21 (*ev21*) and K genes are tightly linked (Bacon et al., 1988; Elferink et al., 2008). Chickens that harbor locus *ev21* express infectious endogenous viruses (EV21; Bacon et al., 1988). The integration of *ev21* is therefore associated with several avian production problems, including reduction in egg production, increases in infection by exogenous avian leucosis virus (ALV; Harris et al., 1984), and increased mortality rates (Smith and Fadly, 1988).

In chickens, exogenous ALVs can be classified into five subgroups including ALV-A, -B, -C, -D, and -J. Compared with endogenous viruses, exogenous ALV, especially subgroup J avian leucosis virus (ALV-J), causes serious commercial losses resulting from neoplasm and immunosuppression (Payne and Nair, 2012; Feng et al., 2016). Endogenous viruses are known to act on exogenous ALV in various ways. There is evidence that *ev21* loci can induce immunological tolerance to exogenous ALV infection (Harris et al., 1984; Bacon et al., 1988). Notably, new virus and disease manifestations can arise via genetic recombination between endogenous viruses and exogenous ALV. It has been well-known that ALV-J caused damage in poultry industry all over the world (Payne and Nair, 2012) and emerged as a result of a recombination event between exogenous ALV and the endogenous retrovirus element designated EAV-HP (Sacco et al., 2000, 2004). In recent years, two novel ALV recombined by ALV-C, ALV-E, and ALV-J were identified in different lines of layer chicken in China (Cai et al., 2013). Furthermore, a novel cross-species recombinant retrovirus has been also reported (Henzy et al., 2014).

Given the continuous rise of labor costs, it seems clear that feathering auto-sexing will be used more and more in the Chinese poultry industry. However, it remains unclear as to whether the presence of *ev21* in late-feathering Chinese yellow chickens has any effect on the host; exogenous ALV infection is of particular concern. Despite extensive control and eradication of programs against exogenous ALV has been put into practice in China, these viruses remain a challenge to Chinese poultry producers. In this respect, it is unknown whether or not a novel and dangerous ALV subgroup that recombined by ev21 and ALV-J will appear, and when the new recombined virus will appear. Thus, it is necessary to continuously monitor the chicken flock harbored *ev21* seriously.

In the present study, two ALV-J strains were isolated from late-feathering chicken flock and fast-feathering chicken flock, respectively. We analyzed the interaction between *ev21* and the ALV-J strain isolated from late-feathering chickens, and further explored the interaction between *ev21* and host innate immune system.

## Materials and methods

### Sample collection

Forty-six 160-day-old, late-feathering Chinese yellow chickens, and the same number of fast-feathering Chinese yellow chickens, were sampled from a farm in Guangdong Province, China.

Blood samples were collected aseptically with anticoagulant and centrifuged at 4°C at 1200 rpm for 15 min to isolate plasma. Plasma samples were stored at −80°C.

All animal procedures were performed according to the regulations and guidelines established by the South China Agriculture University Institutional Animal Care and Use Committee and international standards for animal welfare.

### *ev21* detection

DNA was extracted from the blood of late-feathering and fast-feathering yellow chickens as mentioned above using an NRBC Blood DNA Kit (Omega, USA). Previously published primers were used to screen chickens for the integration of chicken endogenous retrovirus *ev21* (Tixier-Boichard et al., 1994) and described as follows, PR-UP (5′-GTGGGAATGGTACTACAGAGAAGG-3′), PR-DWN (5′-CATTTCAAGCAAGGGACTGGC-3′) and Pl-IN (5′-ACCTGAATGAAGCTGAAGGCTTC-3′).

### Exogenous virus isolation

The aforementioned plasma samples were inoculated into DF-1 cell suspensions (1.75 × 10^5^ cells/well) in 24-well plates (Corning, USA). DF-1 cells, which are known to be susceptible only to exogenous ALV (Himly et al., 1998), were obtained from ATCC (Manassas, VA, USA). The DF-1 cell suspensions with the plasma samples were grown in DMEM supplemented with 10% fetal bovine serum (FBS), at 37°C in the presence of 5% CO_2_. After 24 h of incubation, the supernatant was removed and fresh DMEM medium (1% FBS, 100 U/mL penicillin and 100 mg/mL streptomycin) was added. These DF-1 cells were maintained at 37°C with 5% CO_2_ for 7 days with daily monitoring, after which, the cells were frozen and thawed (three times) and the sample supernatants were harvested. The supernatant collected was tested for ALV group-specific antigen (p27) via an antigen-capture enzyme-linked immunosorbent assay (ELISA) (IDEXX, USA) according to the manufacturer's instructions. The results were expressed as s/p ratios where s/p = (Sample Mean − Kit Negative Control Mean)/(Kit Positive Control Mean − Kit Negative Control Mean). DNA was extracted (Omega, USA) from ELISA-positive samples selected from late- and fast- feathering chicken groups; these samples were tested via PCR with previously published primers for different subgroups of exogenous ALVs (A, B, and J; Smith et al., 1998; Lai et al., 2011).

### Immunofluorescence assay (IFA) and western blot analysis

The two ELISA-positive samples selected from late and fast feathering chicken groups were also evaluated with Immunofluorescence assays (IFA) and Western blotting following our previously described method with ALV-J envelope protein specific monoclonal antibody JE9 (kindly provided by Dr. Kun Qian, Yangzhou University) (Venugopal et al., 1997; Dai et al., 2016a). IFA was performed using the FITC-labeled anti-mouse IgG (Sigma, USA) and analyzed by fluorescence microscope using NIS-Elements BR analysis software (Nikon, Japan). IRDye 800-conjugated anti-mouse IgG or IRDye 700DX-conjugated anti-rabbit IgG (1:10,000; Rockland Immunochemicals, USA) were as the secondary antibody in western blot analysis, and analyzed with an Odyssey infrared imaging system (LI-COR Biosciences, USA).

### Cloning and sequencing of the *ev21* element and ALV-J genomes

The *env* gene and a long terminal repeat (LTR) of *ev21* were amplified with specific primers designed based on the published corresponding sequences (X54094.1); PCR amplification was performed using a blood DNA template from three late- feathering chickens (Table 1).

**Table 1:** **Primers designed in this study**.

**Target**	**Primer**	**Sequence (5′-3′)**	**Accession no**.
ev21env	Forward	gggg**ggtctctagtg**attcccagtcgtccggtag	X54094.1
	Reverse	gccggg**tctcgtggg**ttacactgctctattttcgg	
ev21LTR	Forward	aaatgtagtcaaatagagccag	X54094.1
	Reverse	tgaagccttcagcttcattcag	
M180env	Forward	gggggg**tctctagtg**gaagccgtcataaaggcatttctgactggacac	KX611834
	Reverse	gccggg**tctcgtggg**ctaccgctgctcccaaatt	
M180LTR	Forward	cgg**ggtacc**tgtagtcttatgcaatacg	KX611834
	Reverse	ccc**aagctt**aatgaagccttccgcttcatgc	
CH25H	Forward	aatccagccgcagagctatc	NM_001011688.2
	Reverse	cagctctggagctatcaccg	

*Bold, enzyme cutting site*.

The full-length proviral ALV-J genomes were amplified with previously published primers (Li et al., 2013).

PCR was performed according to the manufacturer's instructions of PrimeSTAR® HS DNA Polymerase with GC Buffer (Takara, Japan). The PCR products were excised from 1% agarose-TAE gels and purified using a DNA gel extraction kit (Omega, USA). The purified PCR products were cloned into the TA vector pMD18-T (Takara, Japan). Three independent clones of each pMD18-T vector were sequenced by the Sangon Biotech (Shanghai) Co., Ltd (Shanghai, China).

### Sequence analysis

Sequence alignments were compared between *ev21* and ALV-J isolated from late-feathering chickens. The nucleotide sequences were aligned using the MegAlign function in the sequence analysis software Lasergene (version 7.10; DNASTAR, Madison, WI, USA). Phylogenetic analysis was performed using MEGA ver.4.01 (Pan et al., 2012). The GenBank accession numbers of ALV-J and endogenous ALV reference strains are summarized as follows: HPRS103 (Accession number: Z46390.1), SCAU-HN06 (HQ900844.1), NX0101 (DQ115805.1), EAV-HP (NC_005947.1), ev21 (X54094.1), SD0501 (EF467236.1), and ev1 (AY013303.1).

### Co-immunoprecipitation (co-IP)

The pflg-ev21env plasmid was constructed by cloning the *env* cDNA of *ev21* from late-feathering yellow chicken into the pCMV-flg vector (Full name: CMV-SP6-3^*^flag-[]-SV40 pA; SIDANSAI, China). The pmyc-M180env plasmid, encoding the envelope protein of exogenous ALV, was constructed by inserting the *env* gene of the M180 strain into pCMV-myc vector (Full name: CMV-SP6-myc tag-[]-SV40 pA; SIDANSAI, China). pCMV-flg and pCMV-myc were used as the empty vector (EV) controls. The primers used in the construction of these plasmids are summarized in Table 1.

The bidirectional Co-immunoprecipitation was divided into two groups. In one group, DF-1 cells were co-transfected with pflg-ev21env and pmyc-M180env, pflg-ev21env and pCMV-myc EV plasmids using rabbit anti-MYC Mab (Sigma, USA). In the other group, DF-1 cells were co-transfected with pflg-ev21env and pmyc-M180env, pmyc-M180env, and pCMV-flg EV plasmids using mouse anti-Flag Mab (Sigma, USA) by Lipofectamine 3000 reagent according to the manufacturer's instructions (Invitrogen, USA). The transfected DF-1 cells were maintained at 37°C with 5% CO_2_. At 24 h post-transfection, these cells were lysed with Cell lysis buffer for Western and IP (Beyotime Inst Biotech, China), and the sample supernatants were obtained by centrifugation at 12,000 × g for 5 min. One hundred microliters of lysate samples were used for western blotting; the remaining sample volume was used for co-IP analysis. The lysates were precleared with protein A/G plusagarose (Santa Cruz, USA) at 4°C for 10 min, and then the supernatant was collected and incubated with mouse anti-Flag Mab or rabbit anti-MYC Mab (Sigma, USA) at 4°C overnight. Next, protein A/G plus-agarose was added to the mixture and incubated at 4°C for 4 h. The supernatant was removed after centrifugation at 14,000 × g for 1 min, and the residue was washed three times with PBS. The residue was resuspended with 2 × loading buffer and boiled at 100°C for 10 min. The supernatant was collected and analyzed via western blotting using mouse anti-Flag MAb (Sigma, USA) and the JE9 monoclonal antibody (substitute for anti-MYC Mab). IRDye 800-conjugated anti-mouse IgG or IRDye 700DX-conjugated anti-rabbit IgG (1:10,000; Rockland Immunochemicals, USA) were as the secondary antibody in western blot analysis, and analyzed with an Odyssey infrared imaging system (LI-COR Biosciences, USA).

### Luciferase reporter gene assay

Plasmid pGL3-LTR was constructed by inserting the LTR of the M180 strain into the pGL3-Basic vector (Promega, USA) using the KpnI and HindIII sites. The LTR primer of the M180 strain is detailed in Table 1. The pRL-TK plasmid (Promega, USA) was used as a control plasmid.

DF-1 cells were co-transfected with pflg-ev21env, pGL3-LTR, and pRL-TK plasmid using Lipofectamine 3000 (Invitrogen, USA). The control was co-transfected with pflg-EV, pGL3-LTR, and pRL-TK plasmid. At 24 h post-transfection, the measurement of reporter luciferase activity was performed using Dual-Luciferase Reporter Assay System according to the manufacturer's directions (Promega, USA). Firefly luciferase activities were normalized based on the activities of Renilla luciferase. Data were representative of the results of three independent experiments, all of which including triplicate replication.

### Analysis interferon-stimulated genes (ISGs) by quantitative real-time PCR (qPCR)

DF-1 cells were transfected with the pflg-ev21env and pflg-EV plasmid using Lipofectamine 3000 (Invitrogen, USA). These cells were infected respectively with 0.1 mL of 10^4^ mL^−1^ 50% tissue culture infective dose (TCID_50_) of ALV-J strain M180 at 24 h post-transfection. Total RNA was extracted from infected cells at 6h and 24 h post infection (hpi) using an RNAfast200 kit (Fastagen, Shanghai, China), followed by cDNA synthesis of mRNA with a PrimeScript RT Reagent Kit (Takara, Japan) according to the manufacturer's protocol. Chicken ISGs including eukaryotic translation initiation factor 2 alpha kinase 2 (*EIF2AK2/PKR*), alpha-inducible protein 27-like 2 (*IFI27L2/ISG12-1*), zinc finger CCCH-type, antiviral 1 (*ZC3HAV1/ZAP*), and cholesterol 25-hydroxylase (*CH25H*) and ALV-J *env* gene (*gp85*) were analyzed using qPCR. The qPCR primers used in this study, with the exception of those for the amplification of *CH25H*, have been reported previously (Dai et al., 2015; Kint et al., 2015; Feng et al., 2016); the primers for CH25H are detailed in Table 1. The GAPDH gene was used as an internal control; template DNA prepared from uninfected DF-1cells was used as a control. qPCR was performed on a Biorad CFX96 Real-Time Detection System using iTaqTM Universal SYBR® Green Supermix Kit reagents (Biorad, CA, USA). Data analyses were performed using the 2^−ΔΔCt^ method (Livak and Schmittgen, 2001). The replication of the M180 strain was evaluated with the JE9 monoclonal antibody at 6 and 24 hpi. The level of envelope protein was analyzed using Image J version 1.48 (National Institutes of Health, USA).

### Statistical analyses

Statistical comparisons were performed using GraphPad Prism 5 (GraphPad Software Inc., USA). Results are presented as means ± SEM, and statistical significance was assessed at *P* < 0.05, 0.01, or 0.001.

## Results

### *ev21* detection

According to the published work (Tixier-Boichard et al., 1994), we know that normal late-feathering chickens give composite patterns consisting of the 390 bp band that is diagnostic of the *ev21* occupied site and the 515 bp unoccupied site band, and that standard fast-feathering chickens produced a single amplified band at about 515 bp, whereas some revertant fast-feathering chickens yielded only the 390 bp fragment that is specific for the presence of *ev21*.

**Figure 1:**
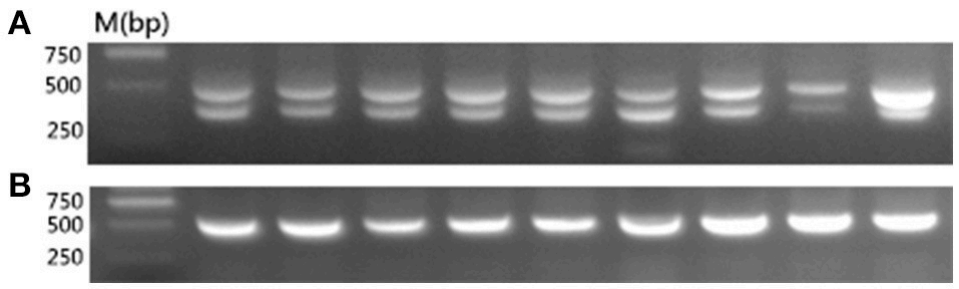
**PCR detection for the ***ev21*** assay. (A)** Late-feathering chickens produced two amplification bands at about 515 and 390 bp. **(B)** Fast-feathering chickens produced a single band at about 515 bp. All samples were detected in this study and the results complied with the above figure. Given the limited space available, this figure shows only partial results.

To determine whether the late-feathering Chinese yellow chickens used in this study carried ev21, we tested for the presence of ev21 via PCR. As expected, all of the late-feathering chickens produced two amplification bands at about 515 and 390 bp (Figure 1A), whereas the fast-feathering chickens only showed the ~515 bp band (Figure 1B). These results confirmed that the late-feathering Chinese yellow chickens used in this study are normal late feathering chickens with the *ev21* insertion, and that the fast-feathering Chinese yellow chickens are standard fast-feathering chickens without the *ev21* insertion.

### ALV-J isolation and identification

**Figure 2:**
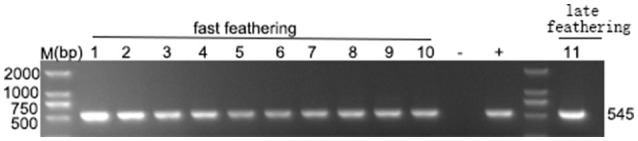
**Detection of ALV-J specific elements**. ELISA positive samples were selected to detect the ALV-J specific 545 bp fragment. Lanes 1–10, fast feathering chicken samples. Lane 11, late feathering chicken sample. All the ELISA positive samples produced specific 545 bp fragment. (−) negative control (purified water), (+) positive control (DNA extracted from the DF-1 cells infected with ALV-J strain SCAU-HN06, Lai et al., 2011).

**Figure 3:**
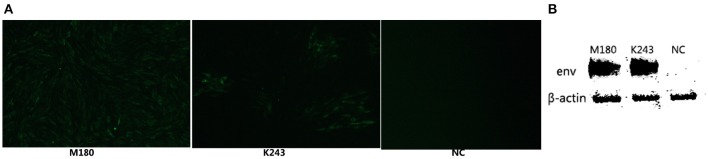
**ALV-J strain identification by IFA and western blot analysis**. The two ALV-J strains isolated from late and fast feathering chicken groups were detected by IFA and Western blot. **(A)** DF-1 cells infected with M180 strain isolated from late-feathering chicken and K243 strain isolated from fast-feathering chicken showing ALV-J specific green fluorescence, 150x magnification. **(B)** DF-1 cells infected with M180 and K243 produced specific ALV-J envelope protein blots. Uninfected DF-1 cells were used as a negative control (NC).

In our study, ELISA results indicated that 10 of the 46 plasma samples were positive (21.7%) in fast-feathering chicken flock and 1 of 46 (2.17%) positive samples in late-feathering chicken flock. Using ALV-J specific primers (Smith et al., 1998), PCR amplification from DNA template extracted from infected DF-1 cells produced a specific 545 bp fragment (Figure 2). The primers for ALV-A/B with same DNA template did not result in the amplification of any specific fragments (data not shown). Two samples were selected for further verification using IFA and western blot analysis. The positive results from the IFA (Figure 3A) and western blot analysis (Figure 3B) demonstrated that the viruses isolated in this study are indeed ALV-J. The two virus stains isolated from late-feathering chicken and fast-feathering chicken were named, respectively, M180 and K243.

### Sequence analysis

The full-length proviral genomes of M180 (GenBank accession number KX611834) and K243 (GenBank accession number KX611833) were sequenced and compared with representative strains of ALV-J [HPRS103 (Z46390.1) and SCAU-HN06 (HQ900844.1)], and endogenous viruses [EAV-HP (NC_005947.1) and ev21 (X54094.1)]. The full-genome sequences of M180 and K243 were 7607 and 7616 bp in length respectively, with a typical of the replication-competent retroviral and without viral oncogenes.

The sequences of M180 and K243 displayed high nucleotide homology (99.3%) with each other. The *env* gene of the two isolates showed 94.2–94.8% nucleotide identity to reference ALV-J strains and EAV-HP. These results indicated that M180 and K243 have a closest phylogenetic relationship with ALV-J.

The gene elements (LTR, *env*) of *ev21* and M180 were amplified using DNA template from the same late-feathering chicken sample. The sequence of *ev21* amplified in this study was the same as the published sequence (X54094.1). Compared with the *env* gene and LTR of *ev21* and the corresponding sequence of M180, the nucleotide identity of LTR was 69.7% and *env* gene was 58.4%, respectively, especially the amino acid identity of *env* gene as low as 14.2%. The results showed that there was no evidence of recombination between M180 and *ev21*. The details of these pairwise comparisons are described in Table 2.

**Table 2:** **Comparison of the nucleotide, amino acid sequences of M180 with the other five ALVs**.

**M180**	**Other ALV strains**				
	**HPRS-103**	**SCAU-HN06**	**K243**	**EAV-HP**	**ev21**
Full-length proviral sequence (7607 bp)	95.8% (7841 bp)	95.3% (7642 bp)	99.3% (7616 bp)	55.9% (4302 bp)	64.6% (1183 bp)
LTR (314 bp)	93.0% (325 bp)	91.7% (325 bp)	100.0% (314 bp)	50.6% (316 bp)	69.7% (277 bp)
*gag* (2106 bp; 701 aa)	96.6% (2106 bp) 97.9% (701 aa)	95.3% (2106 bp) 97.2% (701 aa)	99.7% (2106 bp) 99.6% (701 aa)	53.4% (3375 bp) 44.4% (742 aa)	D
*pol* (2622 bp; 874 aa)	96.9% (2622 bp) 99.0% (874 aa)	97.2% (2622 bp) 98.9% (874 aa)	99.9% (2622 bp) 99.8% (874 aa)	D	D
*env* (1689 bp; 563 aa)	94.7% (1692 bp) 92.5% (564 aa)	94.2% (1692 bp) 91.8% (307 aa)	97.4% (1698 bp) 95.7% (566 aa)	94.8% (1301 bp) 92.4% (433 aa)	58.4% (743 bp) 14.2% (247 aa)

*D, deficiency; aa, amino acid*.

**Figure 4:**
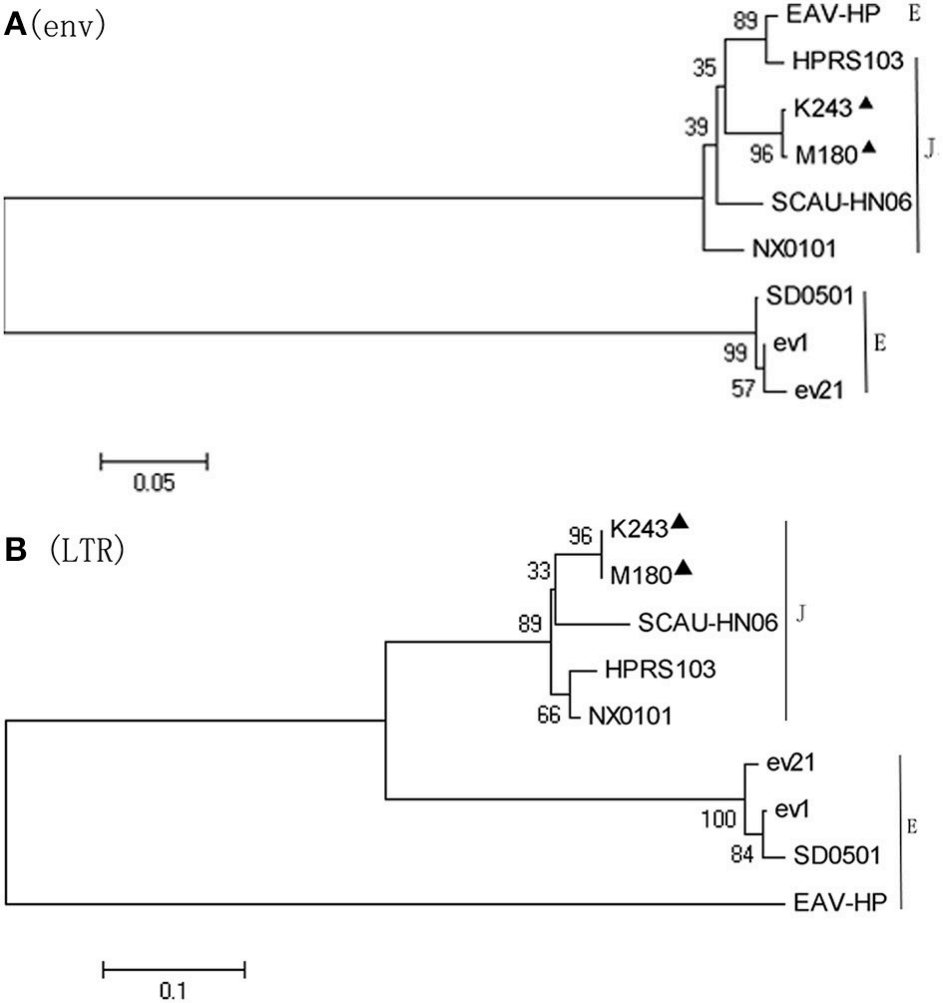
**Phylogenetic analysis based on the ***env*** and LTR sequences**. Phylogenetic relationship of the two novel ALV-J isolates to reference ALV strains and endogenous virus sequences. Black triangles indicate isolates M180 and K243 in this study. **(A)** Phylogenetic analysis of the *env* sequence. **(B)** Phylogenetic analysis of the LTR sequence.

Phylogenetic analysis of the nucleotide sequences of the *env* gene (Figure 4A) and 3′LTR (Figure 4B) demonstrated that M180 was closely related to ALV-J. M180 and *ev21* clustered in a distinct group in the phylogenetic tree of *env* gene and 3′ LTR (Figure 4).

### Interaction of envelope proteins between M180 and *ev21*

**Figure 5:**
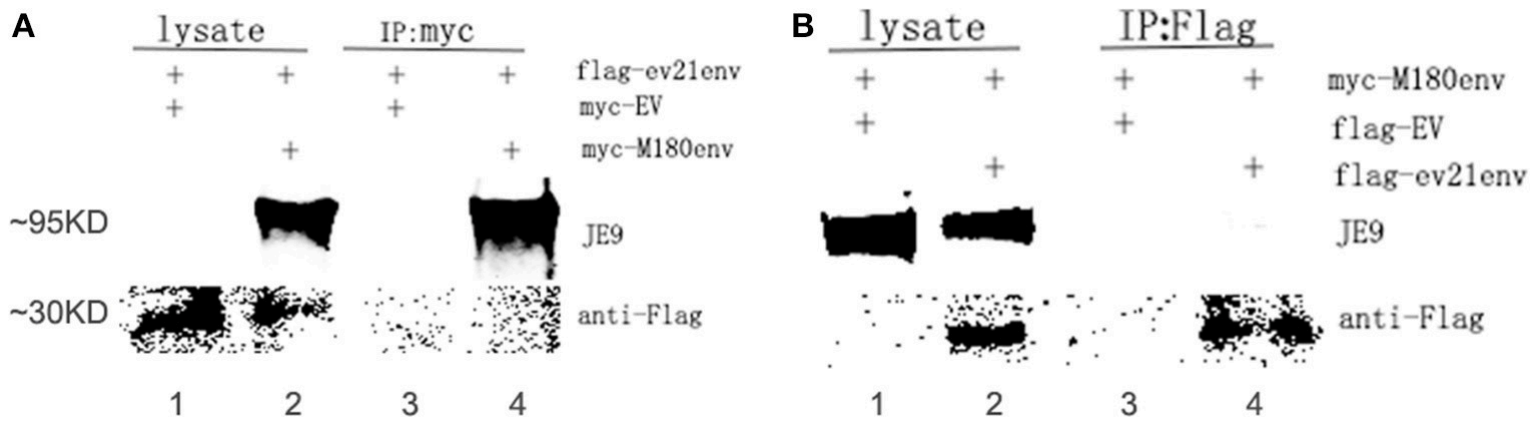
**Analysis of the interaction between M180 strain envelope protein and the ***ev21*** envelop protein. (A)** Co-IP of the M180 envelope protein and the ev21 envelope protein using anti-myc. Co-IP with anti-myc in DF-1 cells co-transfected with pmyc-M180env and pflg-ev21env (lane 2, 4), or with pCMV-myc empty vector (EV) and pflg-ev21env (lane 1, 3). At 24 h post-transfection, cells were lysed and evaluated with western blotting directly (lysate) or co-IP (IP: myc). M180 envelope protein band was separated by 10% SDS-PAGE and detected using ALV-J envelope protein specific monoclonal antibody JE9. The *ev21* envelope protein band was separated by 15% SDS-PAGE and detected using mouse anti-Flag Mab. **(B)** Co-IP of M180 envelope protein and *ev21* envelope protein using anti-Flag. DF-1 cells co-transfected with pflg-ev21env and pmyc-M180env (lane 2, 4), or pCMV-flg empty vector (EV) and pmyc-M180env (lane 1, 3).

A co-IP experiment was performed to confirm the interaction between the ALV-J strain M180 envelope protein and envelope protein encoded by *ev21*. The results of western blot with lysate obtained from the DF-1 cells were co-transfected with pflg-ev21env and pCMV-myc EV plasmids that showed only the specific ev21 envelop protein band (~30 KD; Figure 5A, lane 1), M180 envelop protein (~95 KD), and ev21 envelop protein band produced when co-transfected with pflg-ev21env and pmyc-M180env (Figure 5A, lane 2). This result confirmed that pflg-ev21env and pmyc-M180env function in this system, and that pCMV-myc empty vector control have no protein expression. As expected, there is no protein band detected using anti-myc for Co-IP when co-transfected with pflg-ev21env and pCMV-myc EV plasmids in DF-1 cells (Figure 5A, lane 3). However, the result of Co-IP using anti-myc in lane 4 of Figure 5A showed that M180 envelop protein can be detected but ev21 envelop protein has not been detected in the DF-1 cells co-transfected with pmyc-M180env and pflg-ev21env (Figure 5A, lane 4). This result demonstrated that M180 envelop protein and ev21 envelop protein have no interaction with each other. Furthermore, Co-IP with anti-Flag used in DF-1 cells in the same way mentioned above also demonstrated that M180 envelop protein and ev21 envelop protein have no interaction (Figure 5B).

### Effects of the envelope protein of *ev21* on M180 LTR promoter activation

**Figure 6:**
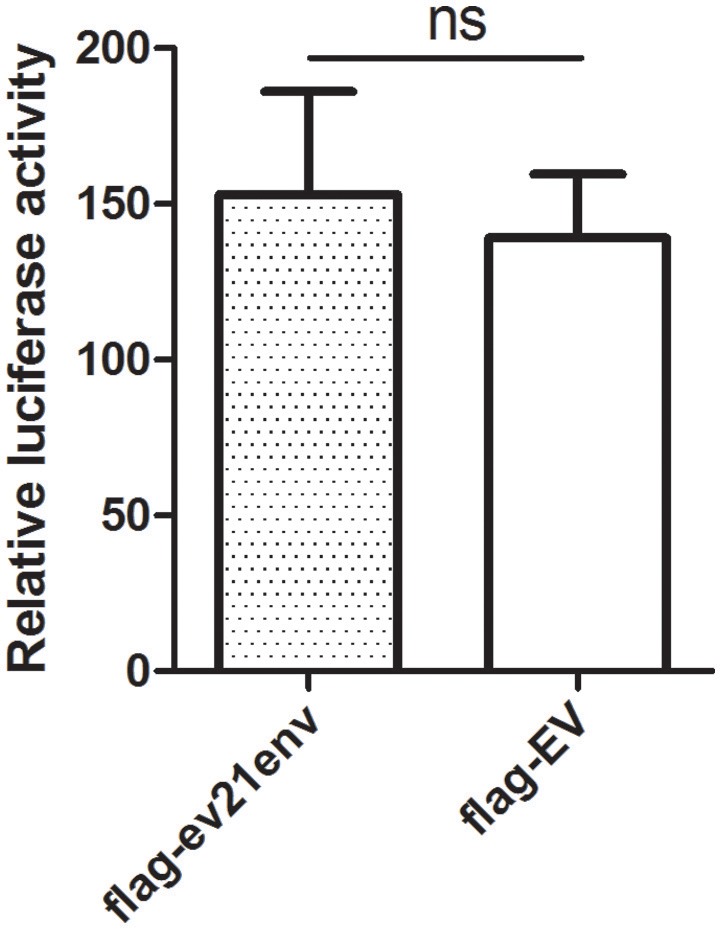
**Influence of the ***ev21*** envelope protein on M180 strain LTR promoter activity**. Luciferase-reporter assay was developed to measure ALV-J strain M180 LTR promoter activity. DF-1 cells were co-transfected with pflg-ev21env, pGL3-LTR and pRL-TK plasmid and the control was co-transfected with pflg-empty vector (EV), pGL3-LTR and pRL-TK plasmid. At 24 h post-transfection, cells were lysed for luciferase detection. ns, not significant.

To further investigate whether the envelope protein of *ev21* has an effect on ALV-J replication, a luciferase-reporter assay was developed to measure ALV-J strain M180 LTR promoter activity. We found that the relative luciferase activity of the M180 strain LTR promoter activity did not differ significantly in the presence of the envelope protein of *ev21* (Figure 6). These results suggest that the ALV-J LTR promoter is not activated by the envelope protein of *ev21*, and this element of endogenous *ev21* does not influence on the replication of ALV-J in DF-1 cells.

### Analysis of the interaction of the envelope protein of *ev21* with host ISGs

**Figure 7:**
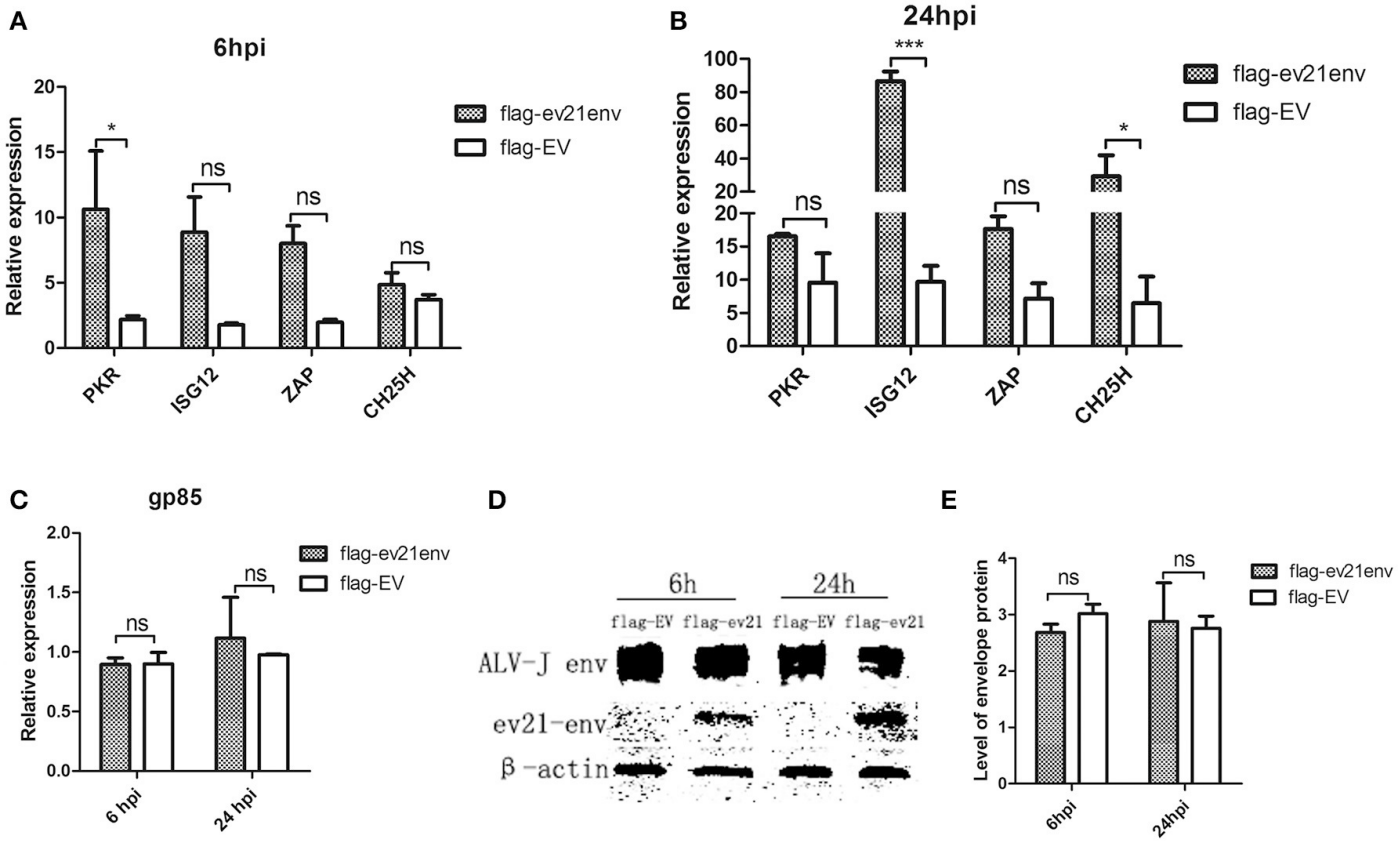
**Analysis of M180 strain replication and ISGs expression**. DF-1 cells transfected with flag-ev21env or flag-EV and infected with M180 strain at 24 h post-transfection. **(A)** Quantitative analysis of the expression of *PKR, ISG12, ZAP* and *CH25H* at 6h post infection. **(B)** Quantitative analysis of the expression of *PKR, ISG12, ZAP* and *CH25H* at 24 h post infection. **(C)** M180 strain production was measured by qPCR at 6 and 24 hpi. **(D)** M180 strain production was measured by Western blot at 6 and 24 hpi. **(E)** The level of M180 strain envelop protein was measured by Image J 1.48. ^*^p < 0.05, ^***^p < 0.001. ns, not significant.

We next explored whether the envelope protein encoded by *env* of *ev21* may rely on host innate immunity to indirectly influence ALV-J replication. Host ISGs expression and ALV-J strain M180 *env* gene expression were analyzed by qPCR and western blot analysis at 6 and 24 hpi. Uninfected and non-transfected DF-1 cells as a control (value is 1, not shown), the expression of all the ISGs detected in our study upregulated in the *ev21* env transfected DF-1 cells compared with the flag-EV transfected group at 6 or 24 hpi (Figures 7A,B). *PKR* expression was significantly higher in the DF-1 cells transfected with flag-ev21env plasmid than that in DF-1 cell transfected with flag-EV at 6 hpi (Figure 7A). And the *ISG12* and *CH25H* expression were significantly upregulated in the *ev21 env* transfected DF-1 cells at 24 hpi (Figure 7B). However, the results showed that ALV-J *gp85* gene expression (Figure 7C) and the level of envelope protein (Figures 7D,E) have no evident differences between *ev21 env* transfected DF-1 cells and the flag-EV transfection group.

## Discussion

ALV are divided into six subgroups including A, B, C, D, E, and J, based on their viral envelope glycoproteins (Payne and Nair, 2012). From 1970 to 1979, ALV-A epidemics occurred frequently while ALV-B was detected rarely, other ALVs were not found in the field (Payne and Nair, 2012). Subsequently, chickens were generally free of avian leucosis caused by ALV until the appearance of the notorious ALV-J in 1987 (Payne and Nair, 2012). ALV-J arose via genetic recombination of exogenous ALV with the endogenous retrovirus EAV-HP *env* sequences (Sacco et al., 2000). Why did a new and great harmful ALV subgroup appear after the eradication of ALV-A/B many years later in some developed countries? What caused the recombination of ALV is an issue for us to ponder over.

Recently, various vaccines against ALV-J have been reported in China (Dou et al., 2013; Xu et al., 2015, 2016). However, there are still no effective vaccines to protect against ALV-J infection in commercial chicken production. Nowadays, China is implementing the work that it has been done in many developed countries to eradicate the ALV. Many large chicken farms are carrying out ALV eradication programs. However, there are different subgroups of ALV and many local chicken breeds, together with the less modernization management in the poultry industry especially among the local chicken breeds, the existence of ALV remain a long time in China. How did ALV-J respond to the pressure of the attacks from humans?

Given that ALV-J prototype virus strain HPRS-103 arose via recombination of exogenous ALV with endogenous retrovirus EAV-HP *env* sequences (Sacco et al., 2004), chicken flocks harbored endogenous retrovirus such as late-feathering chicken (Smith and Fadly, 1988), blue-shelled chicken (Wang et al., 2013), and the recessive white mutation chicken (Chang et al., 2006) should be mainly monitored.

In the present study, ALV-J strains were identified from late-feathering chickens and fast-feathering chickens. Unexpectedly, only one ALV-J strain was isolated from the 46 late-feathering chickens and 10 positive samples were detected among the 46 fast-feathering chickens. The integration of *ev21* was present in all of the late-feathering chickens sampled in this study; this was not found in fast-feathering chickens. Previous studies have shown that late-feathering chickens that harbor *ev21* are susceptible to infection with exogenous ALV (Bacon et al., 1988; Payne and Nair, 2012). We speculate that the results did not meet our expectations due to the small group selected in this study.

We sequenced the genomes of *ev21* and ALV-J strains M180 and K243 and found that there are no similar sequences between M180 and *ev21*. Furthermore, phylogenetic analysis of the *env* gene and the 3′LTR of M180 and *ev21* clustered in a distinct group in a phylogenetic tree. The strong evidence that EAV-HP participated in the recombination of ALV-J strain HPRS-103 was that the EAV-HP *env* element with a high degree (>97%) of sequence identity to the *env* gene of the HPRS-103 (Sacco et al., 2000). Two novel multiple recombinant ALV strains isolated in China displayed high identity with the *gp85* gene in ALV-C, *gp37* gene in ALV-E, and LTR in ALV-J (Cai et al., 2013). Phylogenetic analysis further confirmed that the two ALV strains were recombinant between ALV-J, ALV-E, and ALV-C (Cai et al., 2013). According to our results, we confirmed that recombination did not occur between ALV-J strain M180 and the *ev21* elements in late-feathering Chinese yellow chicken.

LTR regulate ALV replication due to including promoters and enhancers for the growth of ALV (Bizub et al., 1984; Gao et al., 2015). Based on our luciferase assay results, we confirmed that the replication of M180 was not influenced by the envelope protein encoded by the *env* gene of *ev21*. We further confirmed that there was no interaction between the M180 envelope protein and the *ev21* envelope protein with co-IP experiments. Therefore, there is no evidence supporting the idea that an interaction between exogenous ALV and endogenous *ev21* in late-feathering Chinese yellow chicken. We speculate that endogenous *ev21* may exert an effect on exogenous ALV via the host immune system.

Previous study has found that endogenous viruses can induce immunological tolerance to exogenous ALV infection (Bacon et al., 1988). However, endogenous *ev21* may not influence the adaptive immunity in late-feathering and fast-feathering chickens (Bacon et al., 1986). Given that immunological tolerance is not perfect and that endogenous viruses are part of the host genome, *ev21* may activate the innate immune response by producing pathogen-associated molecular patterns (PAMPs) including proteins or nucleic acids (Hurst and Magiorkinis, 2015). Recently, a study published in *Science* showed that endogenous retroviruses have been co-opted to regulate host innate immunity (Chuong et al., 2016). In this study, we also found that the envelope protein of endogenous *ev21* can facilitate the expression of *PKR* at 6h post ALV-J infection and facilitate the expression of *ISG12* and *CH25H* at 24 hpi. Hundreds of ISGs are known to be induced by many viruses, and directly exert antiviral functions (Schoggins and Rice, 2011). However, to date, relatively few chicken ISGs have been identified, and their functions remain poorly understood. Our results, suggest that the envelope protein of endogenous *ev21* was co-opted to regulate the expression of ISGs in the ALV-J infected DF-1 cells. Further studies will be necessary to further characterize their specific action mechanism and to extend our findings to living animal. Interestingly, our results showed that the ALV-J replication was not affected by the envelope protein of endogenous *ev21*. This result is consistent with our luciferase assay results. Our previous study found that chicken interferon alpha (ChIFNα) can restrain ALV replication (Dai et al., 2016b). However, it remains unknown as to which specific ISGs may inhibit the replication of ALV. We suspect that ISGs regulated by the envelope protein of endogenous *ev21* are not the specific ISGs that inhibited the replication of ALV-J. In addition, ALV-J may use some unknown strategies to evade host innate immunity. The function of the endogenous retrovirus *env* gene was focused on placentation and receptor interference (Mi et al., 2000; Dupressoir et al., 2009; Aswad and Katzourakis, 2012). The antiviral functions of endogenous *ev21 env* gene await further study.

According to our results, new recombined ALV strains were not found and ev21 can induce favorable but weak host innate immune response that can't inhibit ALV replication in DF-1 cells. This is a good news for the poultry industry in China. It has been well-known that ALV-J caused damage in poultry industry all over the world and emerged as a result of a recombination event between exogenous ALV and the endogenous retrovirus element designated EAV-HP (Sacco et al., 2000, 2004). What is more interesting, meattype broiler breeders, and broilers had been generally free of leukotic diseases caused by retroviruses from the 1979 to 1987 (Payne and Nair, 2012). And subsequently, ALV-J outbreak and sweep across the entire world. In order to prevent similar tragedies, I think we should continuously monitor the chicken flock harbored endogenous retrovirus seriously, especially in such a big background that chicken farm in China are eradicating the ALV and the virus is faced with enormous pressure. In addition, although endogenous retrovirus is considered to be our enemy, it is useful to us in sometimes. I think we should be aware the enemies and use it for our own purposes. Lastly, I think that our study can be as a model to continuously monitor the chicken flock harbored endogenous retrovirus and eradicate the new recombined virus in the bud.

In summary, there is no evidence of recombination between ALV-J strain M180 and endogenous retrovirus *ev21* in late-feathering Chinese yellow chicken. The envelope protein of endogenous *ev21* can affect the expression of host ISGs, but appears to have no influence on the replication of ALV-J strain M180. The interaction of endogenous retrovirus ev21 and exogenous ALV in late feathering chicken flock will be constantly monitored.

## Author contributions

MF participated in the design of the study, performed the experiments, collected and analyzed data, and drafted the manuscript. MD performed western blot assay. YT, YL, TX, and HL helped with the animal experiment. MS and XZ participated in the design and coordination of the study. All authors read and approved the final manuscript.

### Conflict of interest statement

The authors declare that the research was conducted in the absence of any commercial or financial relationships that could be construed as a potential conflict of interest.

## References

[B1] Aswad A. Katzourakis A. (2012). Paleovirology and virally derived immunity. Trends Ecol. Evol. 27, 627–636. 10.1016/j.tree.2012.07.007 22901901

[B2] Bacon L. D. Fadly A. M. Crittenden L. B. (1986). Absence of influence on immune competence by the sex-linked gene (K) determining slow feathering in white leghorn chickens. Avian Dis. 30, 751–760. 10.2307/1590580 3814012

[B3] Bacon L. D. Smith E. Crittenden L. B. Havenstein G. B. (1988). Association of the slow feathering (K) and an endogenous viral (ev21) gene on the Z chromosome of chickens. Poult. Sci. 67, 191–197. 10.3382/ps.0670191 2837753

[B4] Bizub D. Katz R. A. Skalka A. M. (1984). Nucleotide sequence of noncoding regions in Rous-associated virus-2: comparisons delineate conserved regions important in replication and oncogenesis. J. Virol. 49, 557–565. 6319755 10.1128/jvi.49.2.557-565.1984 PMC255497

[B5] Cai L. Shen Y. Wang G. Guo H. Liu J. Cheng Z. (2013). Identification of two novel multiple recombinant avian leukosis viruses in two different lines of layer chicken. J. Gen. Virol. 94, 2278–2286. 10.1099/vir.0.054239-0 23884361

[B6] Chang C. M. Coville J. L. Coquerelle G. Gourichon D. Oulmouden A. Tixier-Boichard M. (2006). Complete association between a retroviral insertion in the tyrosinase gene and the recessive white mutation in chickens. BMC Genomics 7:19. 10.1186/1471-2164-7-19 16457736 PMC1373650

[B7] Chuong E. B. Elde N. C. Feschotte C. (2016). Regulatory evolution of innate immunity through co-option of endogenous retroviruses. Science 351, 1083–1087. 10.1126/science.aad5497 26941318 PMC4887275

[B8] Dai M. Feng M. Liu D. Cao W. Liao M. (2015). Development and application of SYBR Green I real-time PCR assay for the separate detection of subgroup J Avian leukosis virus and multiplex detection of avian leukosis virus subgroups A and B. Virol. J. 12, 52. 10.1186/s12985-015-0291-7 25889925 PMC4403717

[B9] Dai M. Feng M. Ye Y. Wu X. Liu D. Liao M.. (2016a). Exogenous avian leukosis virus-induced activation of the ERK/AP1 pathway is required for virus replication and correlates with virus-induced tumorigenesis. Sci. Rep. 6:19226. 10.1038/srep19226 26754177 PMC4709637

[B10] Dai M. Wu S. Feng M. Feng S. Sun C. Bai D.. (2016b). Recombinant chicken interferon-alpha inhibits the replication of exogenous avian leukosis virus (ALV) in DF-1 cells. Mol. Immunol. 76, 62–69. 10.1016/j.molimm.2016.06.012 27372921

[B11] Dou W. Li H. Cheng Z. Zhao P. Liu J. Cui Z.. (2013). Maternal antibody induced by recombinant gp85 protein vaccine adjuvanted with CpG-ODN protects against ALV-J early infection in chickens. Vaccine 31, 6144–6149. 10.1016/j.vaccine.2013.06.058 23831319

[B12] Dupressoir A. Vernochet C. Bawa O. Harper F. Pierron G. Opolon P.. (2009). Syncytin-A knockout mice demonstrate the critical role in placentation of a fusogenic, endogenous retrovirus-derived, envelope gene. Proc. Natl. Acad. Sci. U.S.A. 106, 12127–12132. 10.1073/pnas.0902925106 19564597 PMC2715540

[B13] Elferink M. G. Vallée A. A. Jungerius A. P. Crooijmans R. P. Groenen M. A. (2008). Partial duplication of the PRLR and SPEF2 genes at the late feathering locus in chicken. BMC Genomics 9:391. 10.1186/1471-2164-9-391 18713476 PMC2542384

[B14] Feng M. Dai M. Xie T. Li Z. Shi M. Zhang X. (2016). Innate immune responses in ALV-J infected chicks and chickens with hemangioma *in vivo*. Front. Microbiol. 7:786. 10.3389/fmicb.2016.00786 27252695 PMC4879323

[B15] Gao Y. Guan X. Liu Y. Li X. Yun B. Qi X.. (2015). An avian leukosis virus subgroup J isolate with a Rous sarcoma virus-like 5′-LTR shows enhanced replication capability. J. Gen. Virol. 96, 150–158. 10.1099/vir.0.071290-0 25274857

[B16] Harris D. L. Garwood V. A. Lowe P. C. Hester P. Y. Crittenden L. B. Fadly A. M. (1984). Influence of sex-linked feathering phenotypes of parents and progeny upon lymphoid leukosis virus infection status and egg production. Poult. Sci. 63, 401–413. 10.3382/ps.0630401 6326072

[B17] Henzy J. E. Gifford R. J. Johnson W. E. Coffin J. M. (2014). A novel recombinant retrovirus in the genomes of modern birds combines features of avian and mammalian retroviruses. J. Virol. 88, 2398–2405. 10.1128/JVI.02863-13 24352464 PMC3958055

[B18] Himly M. Foster D. N. Bottoli I. Iacovoni J. S. Vogt P. K. (1998). The DF-1 chicken fibroblast cell line: transformation induced by diverse oncogenes and cell death resulting from infection by avian leukosis viruses. Virology 248, 295–304. 10.1006/viro.1998.9290 9721238

[B19] Hurst T. P. Magiorkinis G. (2015). Activation of the innate immune response by endogenous retroviruses. J. Gen. Virol. 96, 1207–1218. 10.1099/jgv.0.000017 26068187

[B20] Kint J. Fernandez-Gutierrez M. Maier H. J. Britton P. Langereis M. A. Koumans J.. (2015). Activation of the chicken type I interferon response by infectious bronchitis coronavirus. J. Virol. 89, 1156–1167. 10.1128/JVI.02671-14 25378498 PMC4300645

[B21] Lai H. Zhang H. Ning Z. Chen R. Zhang W. Qing A.. (2011). Isolation and characterization of emerging subgroup J avian leukosis virus associated with hemangioma in egg-type chickens. Vet. Microbiol. 151, 275–283. 10.1016/j.vetmic.2011.03.037 21632189

[B22] Li Y. Liu X. Liu H. Xu C. Liao Y. Wu X.. (2013). Isolation, identification, and phylogenetic analysis of two avian leukosis virus subgroup J strains associated with hemangioma and myeloid leukosis. Vet. Microbiol. 166, 356–364. 10.1016/j.vetmic.2013.06.007 23876931

[B23] Livak K. J. Schmittgen T. D. (2001). Analysis of relative gene expression data using real-time quantitative PCR and the 2(-Delta Delta C(T)) Method. Methods 25, 402–408. 10.1006/meth.2001.1262 11846609

[B24] Mi S. Lee X. Li X. Veldman G. M. Finnerty H. Racie L.. (2000). Syncytin is a captive retroviral envelope protein involved in human placental morphogenesis. Nature 403, 785–789. 10.1038/35001608 10693809

[B25] Pan W. Gao Y. Qin L. Ni W. Liu Z. Yun B.. (2012). Genetic diversity and phylogenetic analysis of glycoprotein GP85 of ALV-J isolates from Mainland China between 1999 and 2010: coexistence of two extremely different subgroups in layers. Vet. Microbiol. 156, 205–212. 10.1016/j.vetmic.2011.10.019 22101092

[B26] Payne L. N. Nair V. (2012). The long view: 40 years of avian leukosis research. Avian Pathol. 41, 11–19. 10.1080/03079457.2011.646237 22845317

[B27] Sacco M. A. Flannery D. M. Howes K. Venugopal K. (2000). Avian endogenous retrovirus EAV-HP shares regions of identity with avian leukosis virus subgroup J and the avian retrotransposon ART-CH. J. Virol. 74, 1296–1306. 10.1128/JVI.74.3.1296-1306.2000 10627540 PMC111464

[B28] Sacco M. A. Howes K. Smith L. P. Nair V. K. (2004). Assessing the roles of endogenous retrovirus EAV-HP in avian leukosis virus subgroup J emergence and tolerance. J. Virol. 78, 10525–10535. 10.1128/JVI.78.19.10525-10535.2004 15367619 PMC516401

[B29] Schoggins J. W. Rice C. M. (2011). Interferon-stimulated genes and their antiviral effector functions. Curr. Opin. Virol. 1, 519–525. 10.1016/j.coviro.2011.10.008 22328912 PMC3274382

[B30] Smith E. J. Fadly A. M. (1988). Influence of congenital transmission of endogenous virus-21 on the immune response to avian leukosis virus infection and the incidence of tumors in chickens. Poult. Sci. 67, 1674–1679. 10.3382/ps.0671674 2853868

[B31] Smith L. M. Brown S. R. Howes K. McLeod S. Arshad S. S. Barron G. S.. (1998). Development and application of polymerase chain reaction (PCR) tests for the detection of subgroup J avian leukosis virus. Virus Res. 54, 87–98. 10.1016/S0168-1702(98)00022-7 9660074

[B32] Tixier-Boichard M. H. Benkel B. F. Chambers J. R. Gavora J. S. (1994). Screening chickens for endogenous virus ev21 viral element by the polymerase chain reaction. Poult. Sci. 73, 1612–1616. 10.3382/ps.0731612 7816737

[B33] Venugopal K. Howes K. Barron G. S. Payne L. N. (1997). Recombinant env-gp85 of HPRS-103 (subgroup J) avian leukosis virus: antigenic characteristics and usefulness as a diagnostic reagent. Avian Dis. 41, 283–288. 10.2307/1592179 9201389

[B34] Wang Z. Qu L. Yao J. Yang X. Li G. Zhang Y.. (2013). An EAV-HP insertion in 5′ Flanking region of SLCO1B3 causes blue eggshell in the chicken. PLoS Genet. 9:e1003183. 10.1371/journal.pgen.1003183 23359636 PMC3554524

[B35] Xu Q. Cui N. Ma X. Wang F. Li H. Shen Z.. (2016). Evaluation of a chimeric multi-epitope-based DNA vaccine against subgroup J avian leukosis virus in chickens. Vaccine 34, 3751–3756. 10.1016/j.vaccine.2016.06.004 27318415

[B36] Xu Q. Ma X. Wang F. Li H. Zhao X. (2015). Evaluation of a multi-epitope subunit vaccine against avian leukosis virus subgroup J in chickens. Virus Res. 210, 62–68. 10.1016/j.virusres.2015.06.024 26196055

